# Production of fluorescent antibody-labeling proteins in plants using a viral vector and the application in the detection of *Acidovorax citrulli* and *Bamboo mosaic virus*

**DOI:** 10.1371/journal.pone.0192455

**Published:** 2018-02-06

**Authors:** Song-Yi Kuo, Yuan-Chuen Lin, Yi-Chin Lai, Jia-Teh Liao, Yau-Heiu Hsu, Hsiou-Chen Huang, Chung-Chi Hu

**Affiliations:** Graduate Institute of Biotechnology, National Chung Hsing University, Taichung, Taiwan; US Naval Research Laboratory, UNITED STATES

## Abstract

Serological methods are relatively convenient and simple for the detection of pathogens for front-line workers. On-site visualization of the test results plays a pivotal role in the process. However, an efficient, universal labeling agent for antibodies is needed for the development of efficient serological detection tools. In this study, a *Bamboo mosaic virus* (BaMV)-based viral vector was employed to express recombinant proteins, collectively designated GfED, consisting of *Staphylococcus aureus* Protein A domain ED (SpaED) fused to either the N- or C-terminal of an improved green florescent protein (GFP) with or without the coat protein (CP) of BaMV, efficiently in *Chenopodium quinoa*. The GfED in crude leaf extracts could specifically attach to IgG molecules of rabbits and mice, effectively labeling IgG with GFP, emitting green light at 506 nm when excited at 450 nm using simple, handheld equipment. To demonstrate the applicability of GfED in serological assays, we have developed a fluorescent dot blot assay for the rapid detection of *Acidovorax citrulli* (Ac), a bacterial pathogen of cucurbits, and BaMV, a viral pathogen of bamboos. By using the crude extracts of inoculated *C*. *quinoa* leaves expressing GfED as an IgG-labeling agent, the pathogens were easily and quickly detected through uncomplicated operations using simple equipment, with results observable by the naked eye. Examination using fluorescent microscopy and transmission electron microscopy revealed that the GfED subunits may assemble into virus-like particles, which were further involved in the formation of aggregates of GfED-antibody-antigen complexes with the potential for fluorescence signal enhancement. The results suggested that plant-expressed GfED may serve as a promising alternative of IgG-labeling agent for current serological assays.

## Introduction

The damage from plant pathogens is one of the major reasons causing the losses of crops [1]. The development of detection techniques for plant pathogens is an effective strategy to control the spread of pathogens and cut down the losses attributed to plant diseases [2]. To achieve better disease control, pathogens should be detected at the early growing stages or even before the germination or transplanting of the seeds or seedlings, respectively. For front-line workers, serological detection methods are commonly used in the pathogen indexing and disease inspection processes for convenience. However, on-site visualization of the detection results is a critical prerequisite for a successful detection method. To facilitate the observation of the assay results, many antibody-labeling systems have been developed [3], including covalent or non-covalent labeling with fluorophores, gold nanoparticles, enzymes, biotin, haptens, etc. These methods generally involve the conjugation between the labeling agents with antibodies, followed by the removal of the un-conjugated reactants. However, the ratio between the labeling agents and antibodies needs to be optimized to increase the conjugation efficiency and minimize the interference with the antigen-recognition functionality of the antibodies [4]. Therefore, more efficient methods for antibody-labeling are still in need.

An alternative approach is to construct recombinant proteins between antibody-specific ligands and fluorescent proteins, and use such fluorescent ligands to label antibodies in assays. It has been demonstrated that fusion proteins between green fluorescent protein (GFP) and *Staphylococcus aureus* Protein A (SpA) [5–7] or Streptococcal protein G [8] could be successfully applied in western and dot blot assays. However, in these previous studies, the fluorescent ligands were produced in *Escherichia coli*. Recently, plants have emerged as one of the ideal systems for the production of foreign proteins for their relative ease in scaling-up and high productivity [9,10]. In addition, plant viruses have been utilized as vectors for the efficient expression of foreign proteins (for reviews, see [11–13]). Thus, plant viral vectors might serve as an alternative platform for the production of fluorescent antibody-labeling agents in plants.

We have previously developed a plant viral vector based on *Bamboo mosaic virus* (BaMV) and demonstrated the application of such a vector in the expression of vaccine candidates as chimeric virus particles (CVPs) in plants [14–17]. In addition, we have constructed an infectious clone of BaMV, designated pCB-GFP2a-CP, which led to the production of GFP-tagged BaMV CVPs in the inoculated plants [18]. The CVP strategy offers an advantage of displaying multiple copies of target proteins on the surface of CVPs. In this study, the BaMV-based vector was used to express a recombinant protein consisting of the GFP and *S*. *aureus* Protein A domain ED, designated GfED, efficiently in *Chenopodium quinoa*. The plant-made GfED was used in a modified dot-immunobinding assay to facilitate visualization of the antibody-antigen complex on nitrocellulose membranes. The practical application of GfED in the serological assay was further demonstrated in the detection of *Acidovorax citrulli* (Ac) and BaMV, economically important pathogens of cucurbit [19–21] and bamboo [21,22] crops, respectively. It was shown that plant-made GfED mediated by BaMV-based vector offers a feasible alternative for serological detection methods.

## Materials and methods

### Preparation of virus particles, bacterial cells and Ac-infected muskmelon leaves

BaMV virions were purified from the *C*. *quinoa* leaves mechanically inoculated with plasmid pCB-GFP2a-CP (10 ng/ leaf) and the yield was determined as described previously [23]. *A*. *citrulli* Aac31 and *A*. *cattleyae* OAC1 were cultured in King′s B (KB) [24] agar or broth containing 20 mg/ml of nalidixic acid at 30°C for 24–48 hr. *E*. *coli* DH5α was cultured in Lysogeny Broth (LB) [25] at 37°C for 16 hr. The concentration of bacterial cultures was determined by spectrophotometer readings of optical density at 600 nm (OD_600_). For the inoculation of muskmelon leaves, inoculum was prepared by suspending *A*. *citrulli* Aac31 bacterial cells from a 24 hr agar culture in phosphate-buffered saline (PBS, 137 mM NaCl, 2.7 mM KCl, 10 mM Na_2_HPO_4_, 2 mM KH_2_PO_4_, pH 7.4) and the bacterial concentration was adjusted to an OD_600_ of 0.3, ca. 10^8^ colony-forming units (CFU)/ml. Muskmelon seedlings with 5–6 expanded true leaves were used for inoculation by swabbing on both leaf surfaces with a cotton swab saturated with the inoculum amended with 2% of carboxymethyl cellulose. Inoculated seedlings were maintained in an environment suitable for symptom development [26]. Leaves showing typical bacterial fruit blotch (BFB) symptoms were used for the following experiments.

### Polymerase chain reaction (PCR) amplification

**Fig 1:**
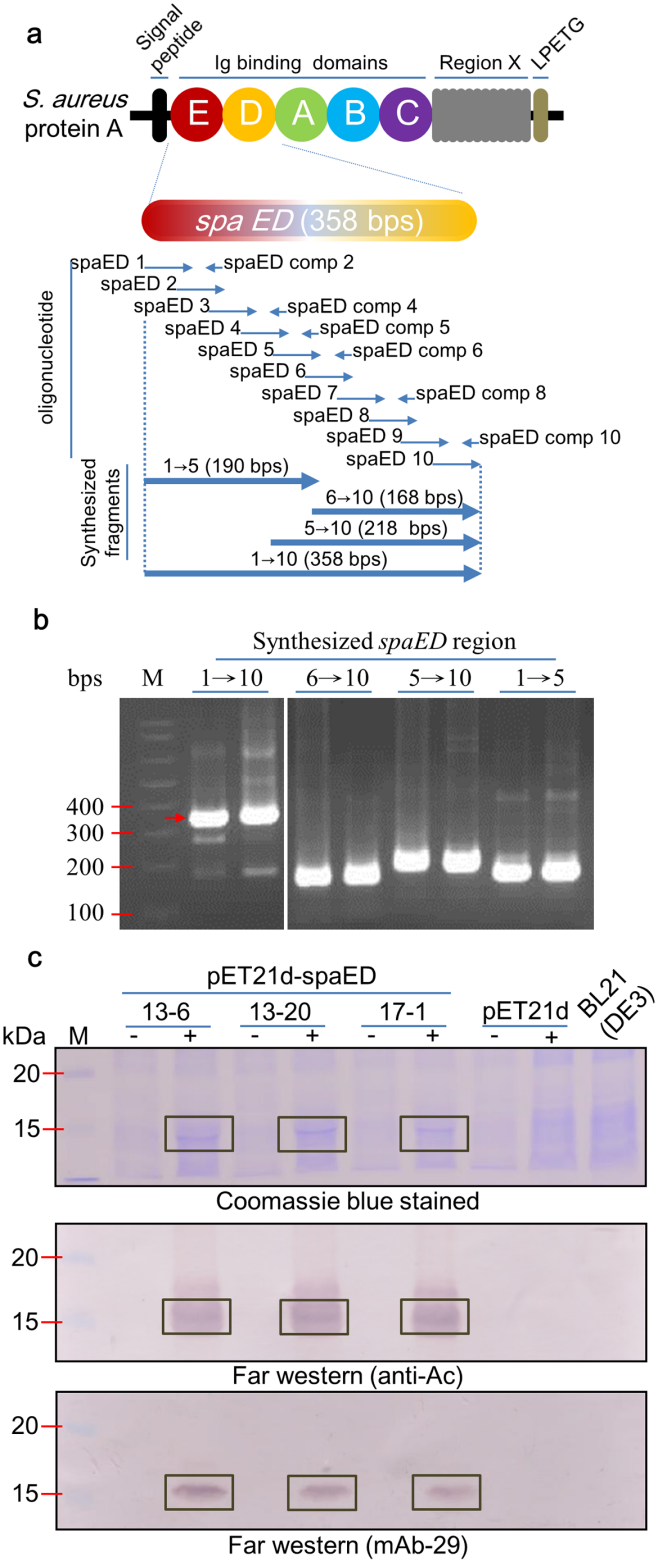
Synthesis of *spaED* and its functionality analysis. **(a)** Schematic illustration of *Staphylococcus aureus* Protein A, *spa* gene. The domain ED was synthesized using 10 forward oligonucleotides (spaED 1 to 10) and 6 reverse complemented oligonucleotides (spaED comp 2, 4, 5, 6, 8, 10). The oligonucleotides were designed with 15-nucleotide overlapping with neighboring oligonucleotides. The expected size of synthesized segments 1→5, 6→10, 5→10, and 1→10 were shown, and verified by electrophoresis through a 1% agarose gel, stained with ethidium bromide, and visualized with UV illumination **(b)**. **(c)** Functionality analysis of over-expressed SpaED protein. The plasmid spaED-pET21d was transformed into *E*. *coli* BL21 (DE3). The expression of SpaED protein was induced by the addition of 1mM IPTG followed by incubation at 37°C for 3 hr. The bacterial cells were then collected by centrifugation at 12000 x*g* for 10 min and lysed by sonication on ice. Samples were subjected to electrophoresis through a 12.5% acrylamide gel containing 1% SDS and stained with Coomassie blue (upper panel). The affinity of SpaED to IgG (anti-Ac and mAb-29) was analyzed by far-western blotting (middle and lower panels, respectively). The clones harboring pET21d-spaED plasmid, 13–6, 13–20, and 17–1, were indicated. pET21d, clone harboring pET21d plasmid; + or -, with or without IPTG induction, respectively; BL21 (DE3), bacterial cells harboring no expression vector and without IPTG induction as negative controls. M, DNA (b) or protein (c) marker.

To obtain the DNA fragment corresponding to SpA domain ED (*spaED*), a PCR-assembly process (Fig 1a) was used to assemble synthetic oligonucleotides designed based on the nucleotide sequence of *Staphylococcus aureus* Protein A (GenBank accession number M18264) [27]. The oligonucleotide and primer sequences used in this study were listed in Table 1. For artificial assembly of *spaED* sequence, oligonucleotides were used as mutual templates, with overlapping and reverse complemented segments as primers in PCR amplification (Fig 1a). The condition used for PCR amplification was as follows: 15 cycles of 94°C for 30 seconds, 55°C for 30 seconds, 66°C for 1 min, followed by 25 cycles of 94°C for 30 seconds, 65°C for 30 seconds, and 72°C for 1 min. The amplified *spaED* fragments were directly cloned into T&A^™^ vector (Yeastern Biotech Co. Taipei, Taiwan), verified by DNA sequencing, and subsequently cloned into proper vectors as described in the following sections.

**Table 1:** Oligonucleotide and primer sequences used in this study.

Name	Sequence^1^	Length (nt)
**Synthesis of *spaED* sequence**		
spaED 1	5’-taccatggcgcaacacgatgaagctcaacaaaatgctttttatcaagtgt-3’	50
spaED 2	5’-ctttttatcaagtgttaaatatgcctaacttaaacgctgatcaacgtaat-3’	50
spaED 3	5’-gctgatcaacgtaatggttttatccaaagccttaaagatgatccaagcca-3’	50
spaED 4	5’-agatgatccaagccaaagtgctaacgttttaggtgaagctcaaaaactta-3’	50
spaED 5	5’-aagctcaaaaacttaatgactctcaagctccaaaagctgatgcgcaacaa-3’	50
spaED 6	5’-gctgatgcgcaacaaaataacttcaacaaagatcaacaaagcgccttcta-3’	50
spaED 7	5’-acaaagcgccttctatgaaatcttgaacatgcctaacttaaacgaagcgc-3’	50
spaED 8	5’-acttaaacgaagcgcaacgtaacggcttcattcaaagtcttaaagacgac-3’	50
spaED 9	5’-agtcttaaagacgacccaagccaaagcactaatgttttaggtgaagctaa-3’	50
spaED 10	5’-tttaggtgaagctaaaaaattaaacgaatctcaagcaccgaaa-3’	43
spaED comp 2	5’-attacgttgatcagcgt-3’	17
spaED comp 4	5’-taagtttttgagcttcac-3’	18
spaED comp 5	5’-ttgttgcgcatcagcttttg-3’	20
spaED comp 6	5’-tagaaggcgctttgttg-3’	17
spaED comp 8	5’-gtcgtctttaagactttg-3’	18
spaED comp 10	5’-tttcggtgcttgagattc-3’	18
**pCB-spaED-GFP2a-CP BaMV viral vector construction**		
N-fus F	5’-taaccggtccatggcgcaacac-3’ (*Age*I)	22
N-fus R	5’-taaccggttttcggtgcttgag-3’ (*Age*I)	22
**pCB-GFP-spaED2a-CP BaMV viral vector construction**		
C-fus F	5’-tagcggccgctaccatggcgcaacac-3’ (*Not*I)	26
C-fus R	5’-tagcggccgctttcggtgcttgag-3’ (*Not*I)	24
**Amplification of inserts within BaMV viral vector**		
5703	5’-aggcatcctatataatatac-3’	20
BCP-N’	5’-ccaaacactaagcagtggat-3’	20

^1^ The restriction enzyme recognition sequences were underlined.

### Plasmid construction

For the verification of the functionality of *spaED* fragment, the plasmid pET21d-spaED was constructed as follows: *spaED* fragment cloned in T&A^™^ vector was digested using *Nco*I and *Bam*HI and cloned into the corresponding sites in pET21d [28] to give pET21d-spaED, which was transformed into *E*. *coli* BL21 (DE3) for T7 RNA polymerase-dependent over-expression. Isopropylthio-β-galactoside (IPTG, 1 mM) induction was performed at 37°C for 3 hr, and the bacterial cells were collected by centrifugation and lysed for further protein analysis. To express the fluorescent tags for antibodies (GfED) in plants, a BaMV-based vector, pCB-GFP2a-CP [18] was used. The *spaED* segments flanked either by artificially designed *Age*I or *Not*I restriction enzyme sites were amplified by PCR using pET21d-spaED as the template with primers listed in Table 1. PCR was performed with the following condition: 35 cycles of 94°C for 30 seconds, 57°C for 30 seconds, and 72°C for 1 min. Following digestion with either *Age*I or *Not*I, the amplified segments were ligated into pCB-GFP2a-CP digested with the corresponding restriction enzyme, resulting in pCB-spaED-GFP2a-CP or pCB-GFP-spaED2a-CP, with SpaED fused to the N- or C-terminus of GFP, respectively. For further PCR amplification of inserts within the above BaMV-based constructs, the condition used was 35 cycles of 94°C for 30 seconds, 55°C for 30 seconds, and 72°C for 1 min.

### Western blotting and far-western blotting

For further verification of the functionality of the GfED, protein extracts from lysed bacterial cells (10^8^ CFU/ml) or 0.1 g leaf tissues of *C*. *quinoa* were subjected to western blot or far-western blot analysis. Protein samples were analyzed by 12.5% polyacrylamide gel electrophoresis containing 1% sodium dodecyl sulfate (SDS-PAGE) [29] or no SDS (native-PAGE). Samples were then transferred [30] from acrylamide gel to PVDF membrane (BioTrace™ PVDF Transfer Membrane, Pall Corporation) and reacted with primary antibodies and a secondary antibody (anti-Rabbit or -Mouse IgG-Alkaline phosphatase antibodies, Sigma Aldrich). The primary antibodies used in this study include those specific to sGFP (anti-GFP) [31], BaMV CP (anti-BaMV CP) [31], bacterial cells or lipo-polysaccharides of Ac (anti-Ac, mAb-29, respectively, Kuo et al. unpublished), and the C4 protein of *Ageratum yellow vein virus* (anti-AYVVC4, Hu et al. unpublished). All of the antibodies were used at a 10000X dilution. For color development, staining solution containing 0.33 mg/ml Nitro blue tetrazolium (NBT), 0.165 mg/ml 5-Bromo-4-chloro-3-indolyl phosphate (BCIP, Promega), 100 mM Tris-HCl, pH 9.5, 150 mM NaCl, and 1 mM MgCl_2_ was applied on membrane for 5–10 min. The staining was terminated by washing with H_2_O.

### Preparation of crude extracts containing GfED fusion proteins and purified virions

The preparation of crude leaf extracts containing GfED and purified virions were performed according to Lin and Chen [23], with slight modifications. *C*. *quinoa* leaves were mechanically inoculated with pCB-GFP2a-CP, pCB-spaED-GFP2a-CP or pCB-GFP-spaED2a-CP plasmid (1 μg/μl, 10 μl per leaf) and harvested at 10 days post inoculation (dpi). The inoculated leaves (100 g) were homogenized in 250 ml TBS (Tris-buffered saline: 50 mM Tris-HCl, pH 7.5, 150 mM NaCl) on ice. After the filtration with 2 layers of cheese cloth, the filtrate was centrifuged at 12000 x*g* for 10 min to collect the supernatant. K_2_HPO_4_ and CaCl_2_ were added drop-wise subsequently to the supernatant to reach the final concentration of 0.04 M each. The mixture was stirred on ice for 10 min and then centrifuged at 12000 x*g* for 10 min to collect the supernatant containing GfED fusion proteins. The supernatant was stored at -20°C until used in the following assays. For further purification of virions, Triton X-100 and PEG 6000 were added to the supernatant to a final concentration of 2% and 6%, respectively. The mixture was stirred for 30 min at 4°C and then centrifuged at 12000 x*g* for 10 min. The pellet was resuspended in TBS buffer and centrifuged in sucrose cushion (20% W/V) at 43000 rpm in a Ti70 rotor (Beckman) for 1 hr at 4°C. The purified virions were finally resuspended in de-ionized water (DI) and stored at -20°C.

### Dot blot analysis

To prepare GfED-tagged antibodies, crude extracts containing GfED were incubated with specific antibodies, including anti-BaMV CP, anti-Ac (polyclonal antibody against Ac), and mAb-29 (monoclonal antibody against Ac) at 4°C for 2 hr and stored at -20°C until use. Samples of extracts from Ac inoculated leaves (0.1 g/ml), Ac bacterial cells (with 5-fold serial dilutions starting at 10^9^ CFU/ml), BaMV viral particles (with 5-fold serial dilution starting at 1000 μg/ml) and the antibodies above were diluted in TBS as indicated in the legend. Samples were loaded (100 μl/well) and coated on nitrocellulose (NC) membrane using Minifold I 96-Well System (GE Healthcare). For the NC membrane coated with samples from leaf extracts, bacterial cells and BaMV viral particles, the membranes were blocked with 0.5% nonfat milk, and overlaid with GfED-tagged antibody (80 μg antibody mixed with 10 ml crude extracts containing GfED) at room temperature for 1 hr. For the NC membrane coated with anti-Ac, the membrane was blocked with lysate of *E*. *coli* BL21 (DE3) cells harboring pET21d-spaED induced with 1 mM IPTG to overexpress SpaED protein. The membrane was then sequentially overlaid with 10 ml of samples and GfED-tagged anti-Ac or mAb-29 at room temperature for 1 hr, and examined under blue light (450 nm) as described below. Alternatively, alkaline phosphatase-conjugated secondary antibodies were used to verify the presence of antibodies, with traditional color development using NBT and BCIP.

### Fluorescent excitation and photography

Samples on native-polyacrylamide gel and NC membrane were observed by illumination with a homemade, handheld LED flashlight which emits blue light (450 nm) as an excitation source, with an orange transparent acrylic pad as the filter. Photographs of the results were taken with an aperture of f/2.5 and the exposure time of 1/13 seconds for pictures of inoculated leaves, 1/40 seconds for native-polyacrylamide gel, and 1/200 seconds for NC membrane, respectively.

### Confocal microscopy

Specific GfED-tagged antibody against Ac was prepared as described above, and then incubated with 1 ml of *A*. *citrulli* Aac31 or *A*. *cattleyae* OAC1 (10^7^ CFU/ml), respectively, at 4°C for 1 hr. After the incubation, bacterial cells were collected by centrifugation at 6000 x*g* for 2 min and washed in TBS for three times. The resulting bacterial pellets were suspended in TBS (100 μl) and applied onto a glass slide. The localization of green fluorescent signal and aggregated bacterial cells were visualized by confocal laser scanning microscopy (Olympus FV1000, Tokyo). The green fluorescence signals were excited and detected at 488 and 525 nm, respectively.

### Transmission electron microscopy (TEM)

The fluorescent BaMV virions (purified from leaves inoculated with pCB-GFP2a-CP) and GfED virus-like particles (VLPs) were examined using immunosorbent TEM. The grids (Formvar/Carbon 200 mesh Cu, Agar Scientific) were coated with the first antibody, anti-BaMV CP (100X dilution), for 20 min. Unbound anti-BaMV CP antibodies were removed by DI washing for 5 min. The grids were subsequently incubated with purified fluorescent BaMV virions or GfED VLPs for 20 min, followed by washing with DI. The anti-GFP and anti-AYVVC4 antibodies (100X dilution) were separately pre-incubated with Goat-anti-rabbit IgG conjugated with gold particles (15 nm in diameter) (anti-Rb Gold, 100X dilution, Agar Scientific) overnight at 4°C, and used to interact with the captured virions on grids for 20 min. The grids were then washed in DI to remove unbound antibodies and the specimens were negatively stained with 1% uranyl acetate (UA) for 5 min. For the bacterial specimens, anti-BaMV CP antibody (100X dilution) was pre-incubated with anti-Rb Gold (100X dilution). Aac31 and OAC1 bacterial cells (10^8^ CFU/ml) were incubated with anti-Ac and GfED at room temperature for 1 hr. The bacteria cells were collected using centrifugation and the unbound anti-Ac or GfED were washed away in DI for three times. The bacteria cells were then resuspended in DI and incubated with anti-Rb Gold linked anti-BaMV CP. The excess antibodies were washed away in DI for three times as mentioned above. The samples were finally mounted on grids and stained with 1% UA for 5 min. The specimens were examined using JEM-1400 (Jeol, Tokyo, Japan).

## Results

### SpaED protein overexpression and functionality analysis

*S*. *aureus* Protein A consists of five immunoglobulin (Ig)-binding domains [32,33], designated E, D, A, B, and C in order, each of which possesses two binding regions with consensus sequence. To avoid the risk of contamination by *S*. *aureus* in the working laboratory, a PCR-based artificial assembly strategy was used. Oligonucleotides were designed based on the sequence of the E-D domain of *spa* gene (GenBank accession number M18264), which are the first and second domains from the N-terminus of the five Ig binding domains. The DNA sequence of *spaED* was synthesized using 10 forward and 6 reverse-complemented oligonucleotides (Fig 1a), each with a 15-nucleotide overlapping region with the neighboring oligonucleotides. The *spaED* coding sequence was assembled by mixing the overlapping and complementary oligonucleotides sequentially in PCR as illustrated in Fig 1a. The results of agarose gel electrophoresis analysis of the final PCR products showed the coding sequence of *spaED* was successfully amplified, with the expected size of 358 bps (Fig 1b, lane 1→10). The nucleotide sequence of the synthesized *spaED* fragment was further confirmed by DNA sequencing. The synthesized *spaED* was cloned into the protein expression vector pET-21d to generate pET21d-spaED, which was transformed into *E*. *coli* BL21 (DE3). The expression of SpaED was examined by SDS-PAGE, followed by Coomassie blue staining. As shown in Fig 1c, upper panel, clones 13–6, 13–20, 17–1 successfully expressed SpaED protein following IPTG induction. To verify the IgG-binding functionality of the expressed SpaED, a far-western blot assay was performed. The total proteins from each clone were separated by SDS-PAGE, and transferred to PVDF membrane. The membranes were incubated with anti-Ac or mAb-29 for 1 hr, followed by the incubation with alkaline phosphatase-conjugated secondary antibody for another 1 hr before color development using NBT/BCIP. If the bacterial cells expressed functional SpaED protein, the IgG molecules would be bound by the SpaED on the PVDF membrane and be detected by the secondary antibody. Results of the far-western blot analysis showed the SpaED expressed in the three clones above had affinity to different antibodies from rabbit (anti-Ac) and mouse (mAb-29), and with the expected molecular weight (around 13 kDa) (Fig 1c, middle and lower panels, respectively).

### Construction of fluorescent SpaED expression viral vector

**Fig 2:**
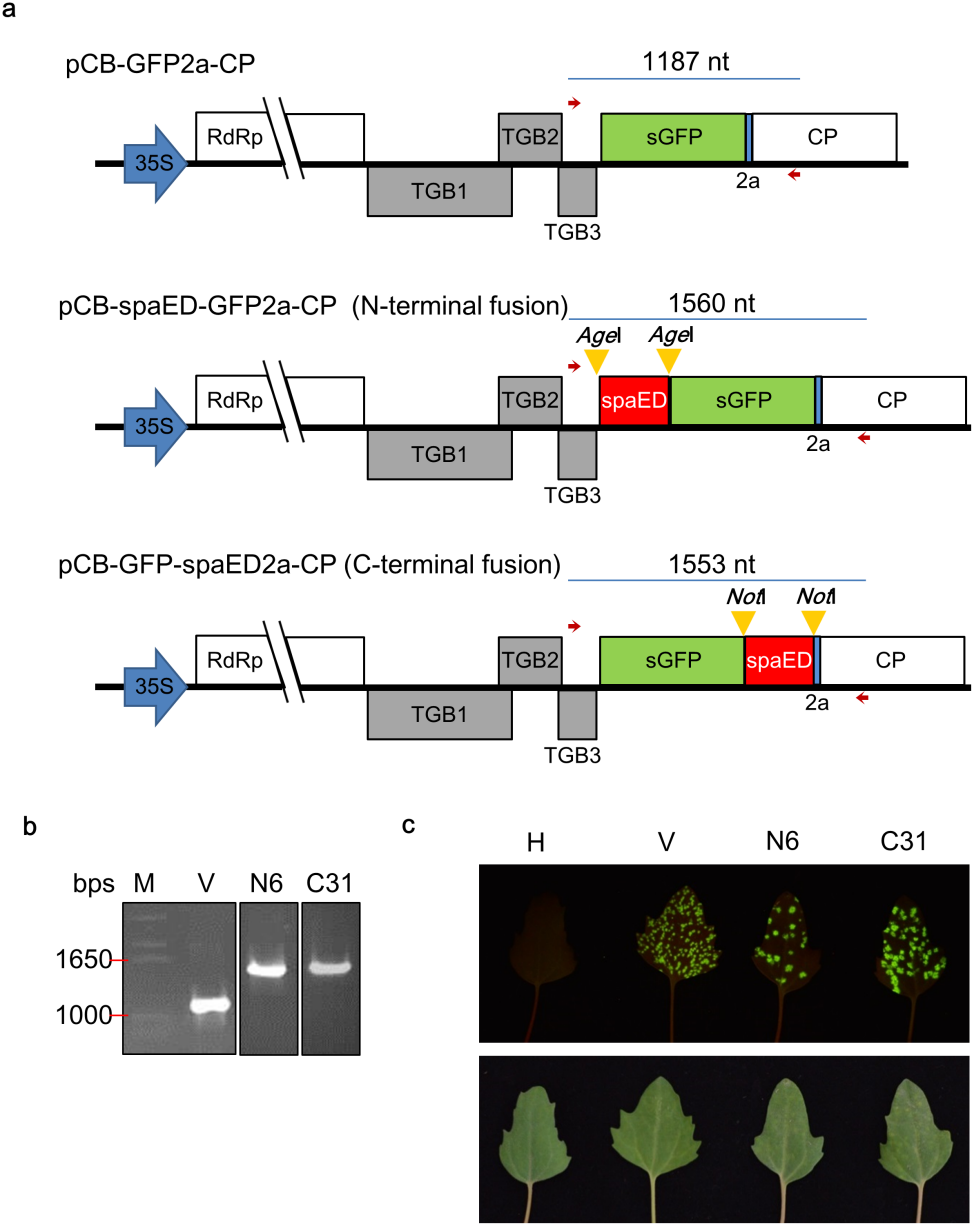
Construction of GfED expression viral vector. The *spaED* sequence was cloned into the BaMV-based viral vector, pCB-GFP2a-CP. **(a)** Schematic diagram showing the viral vector pCB-GFP2a-CP and the construction of GfED fusion protein expression plasmids pCB-spaED-GFP2a-CP (N-terminal fusion) and pCB-GFP-spaED2a-CP (C-terminal fusion). The number above fusion sequence indicated the expected length amplified by colony PCR with specific primers 5703 and BCP-N’ (represented by red arrows) in panel **b**. **(b)** Confirmation of clones by colony PCR. Two selected clones (N6 and C31) were transformed into DH5α and screened by colony PCR and agarose gel electrophoresis. The results showed clones N6 (N-terminal fusion) and C31 (C-terminal fusion) harbored the insert with expected length, as compared to the vector alone (V). M, DNA marker. **(c)** Confirmation of infectivity of the constructs. *Chenopodium quinoa* leaves were inoculated with the plasmid DNAs of clone N6 or C31 (with concentration of 1 μg/μl, 10 μl per leaf). At 10 days post inoculation (dpi), the inoculated leaves were observed under 450 nm blue light with orange filter (upper panel) or under daylight without filter (lower panel). H, healthy leaf; V, leaf inoculated with pCB-GFP2a-CP vector as a positive control.

To take the advantage of plant production system, we adopted the plant viral vector developed previously in our group, BaMV-based vector, pCB-GFP2a-CP [18] to express recombinant proteins consisting of superfolder GFP (sGFP) [34] and the above SpaED (GfED) as a universal labeling agent for antibodies used in serological detection methods. It was expected that sGFP domain could bind to IgG through the SpaED domain, and the target recognized by IgG could be visualized by fluorescence of sGFP. The above *spaED* sequence was cloned into pCB-GFP2a-CP as either the N- or C-terminal fusion to sGFP using *Age*I or *Not*I restriction enzyme sites (yellow arrow head), respectively (Fig 2a), to generate pCB-spaED-GFP2a-CP and pCB-GFP-spaED2a-CP. The constructs were transformed into *E*.*coli* DH5α, and the results were analyzed by colony PCR, using primers 5703 and BCP-N’, which corresponds to the C terminus of TGBp3 and the N terminus of CP, respectively. PCR products with the expected size were amplified from the N terminal fusion clone, N6, and C terminal fusion clone, C31 (Fig 2b). The fusion sequences of *spaED* at both termini of sGFP gene were verified by DNA sequencing. To test the infectivity of the constructs, *C*. *quinoa* leaves were inoculated with the plasmid DNAs of N6 and C31 (10 μg per leaf). By the illumination of a handheld device, BG132 (S1 Text and S1 Fig), the green fluorescent lesions appeared at about 10 days post-inoculation (dpi) (Fig 2c). N6- and C31-inoculated leaves showed fewer lesions than the leaves inoculated pCB-GFP2a-CP, which may be caused by the interference of longer fusions on CP. Nevertheless, the infectivity of the clones in *C*. *quinoa* was confirmed.

### GfED fusion protein expressed in *C*. *quinoa* leaves

**Fig 3:**
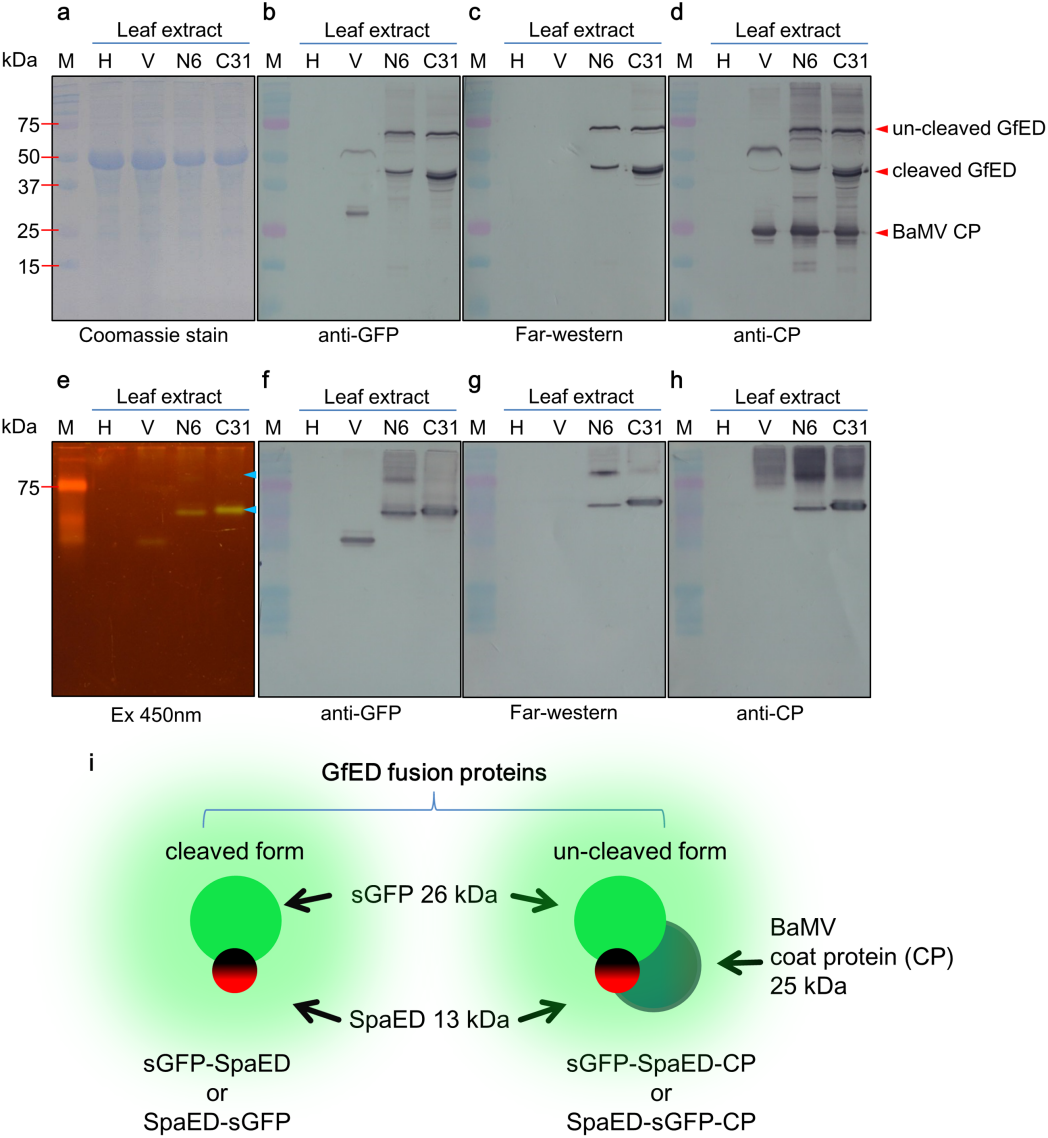
Functional GfED fusion proteins were expressed in inoculated *C*. *quinoa* leaves. Crude extracts from *C*. *quinoa* leaves (0.1 g leaves in 250 μl extraction buffer) were analyzed by SDS-PAGE **(a-d)** or native-PAGE **(e-h)**. Following electrophoresis, the gels were stained with Coomassie blue **(a)** or observed under 450 nm excitation with orange filter **(e)**. Western blot analysis were performed using anti-GFP **(b, f)**, anti-BaMV CP **(d, h)**. For far-western blot analysis, anti-AYVVC4 antibodies were used to probe Protein A **(c, g)**. **(i)** Schematic diagram of the two types of GfED fusion proteins (with or without CP fusion), with molecular weight of each domain indicated. Red arrow heads indicate three structural elements with expected size. Blue arrow heads indicate the fusion protein consisting of sGFP and SpaED domain. H, healthy leaf; V, leaves inoculated with pCB-GFP2a-CP as a positive control; N6, leaves inoculated with pCB-spaED-GFP2a-CP; C31, leaves inoculated with pCB-GFP-spaED2a-CP; M, protein marker.

To verify the expression of functional GfED in the inoculated *C*. *quinoa* leaves, crude extracts from inoculated leaves were analyzed by SDS-PAGE and western blotting (Fig 3). The pCB-GFP2a-CP vector contains a co-translational dissociation peptide, 2a, of *Foot-and-mouth disease virus* (FMDV) at the N-terminus of CP (Fig 2a), which would lead to an incomplete “cleavage” of the fusion proteins at the 2a and CP junction site. Thus the expressed GfED fusion proteins may contain two components (SpaED and sGFP) or three components (SpaED, sGFP, and CP), as shown in Fig 3. As expected, sGFP and BaMV CP in the fusion protein were identified by the western blotting with specific antibodies, anti-GFP and anti-BaMV CP, respectively (Fig 3b and 3d). In addition, the functional SpaED domain was identified by far-western blotting, in which the blot was overlaid with anti-AYVVC4 (antibody against the C4 protein of *Ageratum yellow vein virus*, which is not supposed to recognize the BaMV CP or GFP) (Fig 3c and 3g). Several types of proteins were expected to be expressed from the fusion construct N6 or C31 (Fig 3i): SpaED-sGFP-CP or sGFP-SpaED-CP fusion protein (62 kDa), SpaED-sGFP or sGFP-SpaED fusion protein (37 kDa), and free BaMV CP (25 kDa), all of which were observed in the crude extracts of the inoculated *C*. *quinoa* leaves, as indicated by red arrow heads in Fig 3d. The bands with an estimated molecular weight of 37 kDa on western blotting probed with anti-BaMV CP were considered to be caused by the affinity of SpaED domain of the SpaED-sGFP and sGFP-SpaED fusion protein to anti-BaMV CP IgG (Fig 3d). The signals observed on the membrane probed with anti-GFP may also be resulted from the SpaED domain affinity to anti-GFP IgG. To verify the identity of the protein band, we further analyzed the proteins in the crude extracts of inoculated *C*. *quinoa* leaves using native-PAGE and western blotting. The results showed that two green fluorescent signals were observed in the native-PAGE and matched the corresponding signals in western blot analysis with specific antibodies (Fig 3e and 3f). For practical applications, all of the fusion proteins, including SpaED-sGFP-CP, sGFP-SpaED-CP, SpaED-sGFP, and sGFP-SpaED, may be used to label IgG molecules. Accordingly, the fusion proteins mentioned above were collectively termed as GfED fusion proteins in this study and used un-separated for the following experiments. The detection sensitivity of GfED in crude saps were determined to be 0.4 μg IgG by a dot blot assay similar to that described in Prachayasittikul et al. [7] (S2 Fig). By quantification of specific protein bands using the Multi Gauge version 3.0 software (Fujifilm Life Sciences), the yield of GfED was estimated to be about 0.13 and 0.17 mg/g fresh leaf in N6 and C31 inoculated plants, respectively. The yields were comparable to, or better than, those of our previous studies [15, 16] and the other plant virus based expression system, e.g., 2.4 mg/g fresh leaf tissue for the expression of hepatitis B virus core antigen (HBcAg) virus-like particles (VLPs) or 0.8 mg/g fresh leaf tissue for the expression of Norwalk virus VLPs using TMV based expression systems in *Nicotiana benthamiana* plant [35, 36].

### Detection of virus and bacteria with dot blot analysis

**Fig 4:**
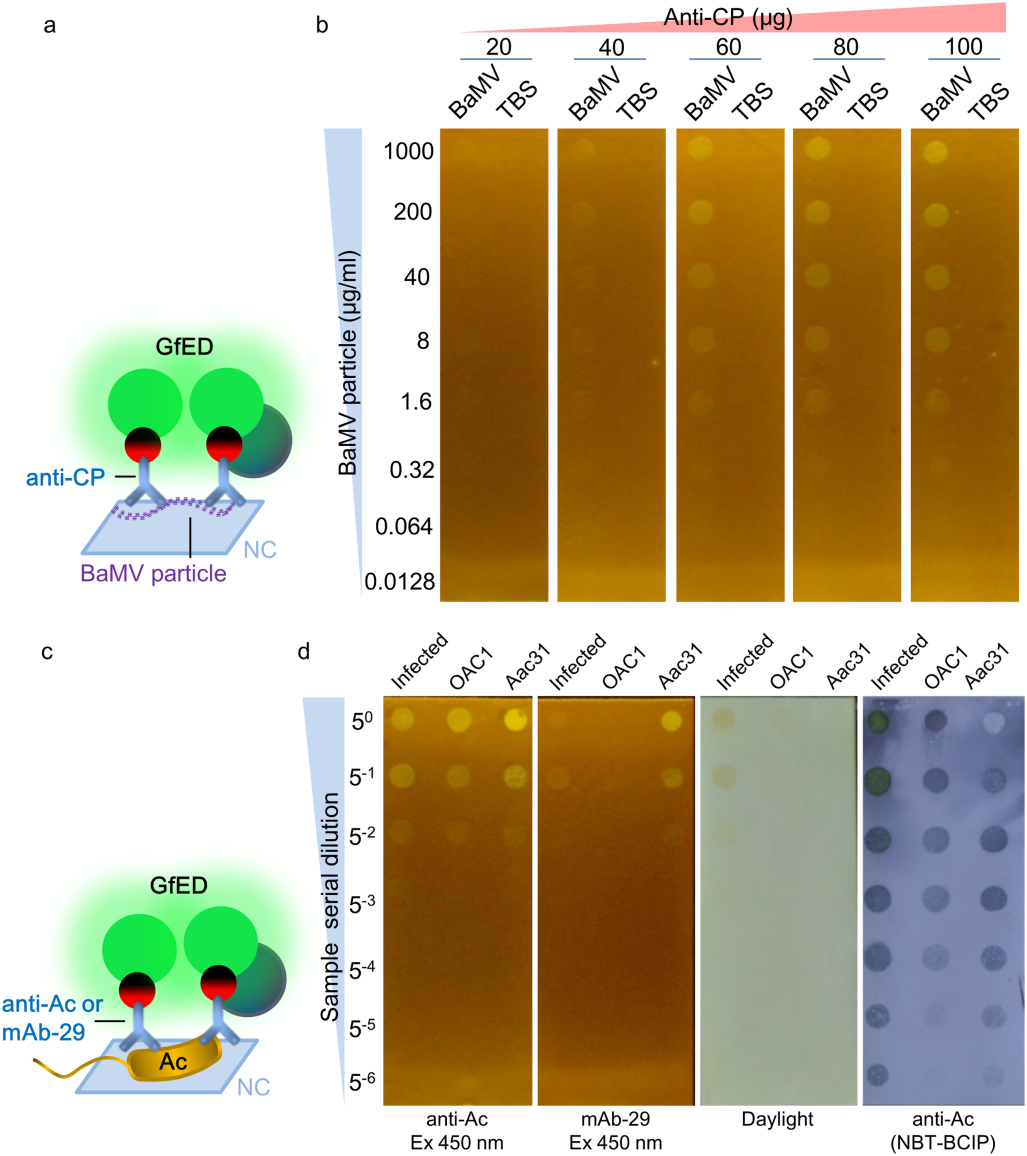
Detection of BaMV and Ac by dot blot analysis using GfED-tagged antibodies. **(a)** Schematic diagram (not drawn to scale) of the proteins involved in panel **b**. **(b)** BaMV particles with 5-fold serial dilution (starting at 1000 μg/ml) were loaded (100 μl/well) on nitrocellulose (NC) membrane and overlaid with GfED, then various amounts of anti-BaMV CP IgG (20, 40, 60, 80, and 100 μg) were added and incubated for 2 hr. The results were observed under 450 nm blue light excitation with an orange filter. BaMV, purified viral particles of BaMV; TBS, NC membrane overlaid with TBS buffer as a negative control. **(c)** Schematic diagram of the proteins involved in panel **d**. **(d)** Bacterial samples with 5-fold serial dilution (starting at 10^9^ CFU/ml) were loaded (100 μl/well) on NC membrane and overlaid (incubated) with 10 ml GfED tagged anti-Ac or mAb-29 (80 μg). The results were examined under the illumination of blue light (450 nm excitation), or daylight, and verified by using traditional dot-blotting with anti-Ac and alkaline phosphatase-conjugated anti-rabbit IgG (anti-Rb AP) antibody, followed by color development with NBT/BCIP. Infected, crude extracts from muskmelon leaves infected with Ac (0.1 g leaf tissues in 1 ml TBS as the starting material); Aac31, *A*. *citrulli* Aac31; OAC1, *A*. *cattleyae* OAC1.

To test the applicability of GfED, we explored the possibility of using GfED in dot blot serological tests for BaMV and Ac, which causes bamboo mosaic on bamboos and bacterial fruit blotch (BFB) diseases on cucurbits, respectively. As an initial step, purified BaMV and cultured Ac were assayed. To simplify the operations in the assay, GfED were pre-incubated with anti-BaMV CP, anti-Ac, or mAb-29 to yield GfED-tagged antibodies, which eliminated the need for the sequential addition of the primary and secondary antibodies. The serial dilutions of purified BaMV virions (starting from 1000 μg/ml) were applied on nitrocellulose (NC) membrane and probed with the GfED-tagged anti-BaMV CP IgG, as illustrated in the schematics shown in Fig 4a. As shown, the BaMV particle with the concentration higher than 64 ng/ml could be detected by the visible fluorescence (Fig 4b). The sensitivity is good enough to detect BaMV in naturally infected plants, which reaches about 0.1 mg/g fresh leaf. For economic considerations, we used the concentration of 80 μg antibody mixed in 10 ml GfED for the following experiments in this study. For the detection of bacterial pathogens, the bacterial sample of *A*. *citrulli* Aac31 and *A*. *cattleyae* OAC1 (non-pathogenic on cucurbits as a negative control) were applied on NC membrane and probed with the GfED-tagged anti-Ac or mAb-29 IgG. *A*. *cattleyae* OAC1 could be recognized weakly by the polyclonal antibody anti-Ac, but not by monoclonal antibody mAb-29 (Kuo et al. unpublished). To test the usability on field samples, the crude extracts of leaves infected with Ac was included in the assays with similar treatment, as indicated in the schematic diagram of the process shown in Fig 4c. The results showed that the Ac could be detected at the concentration of 4×10^6^ CFU/ml, which could be reached at about 3–4 days post inoculation under optimal condition for BFB symptom development, whereas the *A*. *cattleyae* OAC1 could only be barely seen as visible fluorescence on the membrane probed with GfED-tagged mAb-29. The Ac pathogen in samples from inoculated leaves can be detected similarly on membranes probed with anti-Ac or mAb-29 (Fig 4d). The results were verified by using traditional dot blot with anti-Ac and alkaline phosphatase-conjugated goat-anti-rabbit antibody, followed by color development with NBT/BCIP. These results demonstrated that GfED-tagged antibodies could be used to detect viral and bacterial pathogens for the pathogen indexing purposes.

### A “sandwich-type” dot-blotting improves the usability of fluorescent detection technique

**Fig 5:**
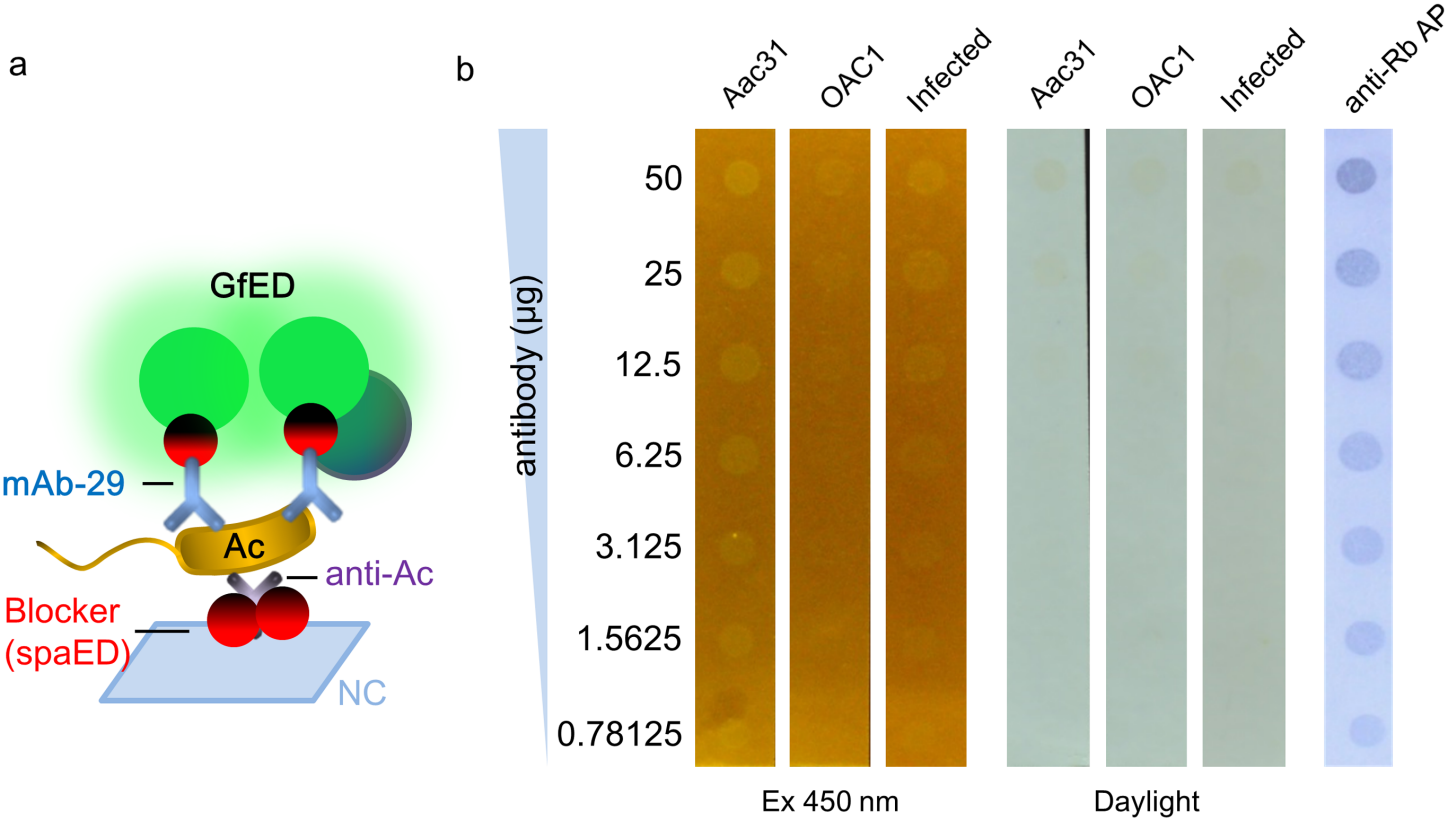
A “sandwich-type” dot-blotting improves the usability of fluorescent detection technique. To improve the sensitivity and specificity, both polyclonal and GfED-tagged monoclonal antibodies were used in a “sandwich-type” dot blot assay. **(a)** Schematic diagram (not drawn to scale) of the “sandwich-type” dot blot assay. **(b)** The samples of bacteria (10^8^ CFU/ml) and crude extracts of Ac-inoculated muskmelon leaves were incubated with NC membrane pre-coated with anti-Ac at room temperature for 1 hr, then washed in TBS. The GfED-tagged mAb-29 was subsequently added and incubated at 4°C for 1 hr. The results were observed under daylight or the illumination of a handheld LED flashlight with excitation wavelength at 450 nm with an orange filter. Serial dilutions of coated IgG were verified by dot-blotting using anti-Rb AP as indicated. Aac31, *A*. *citrulli* Aac31; OAC1, *A*. *cattleyae* OAC1; Infected, sample from the leaves infected with Ac.

To improve the user-friendliness and the specificity of the detection technique above, we devised a “sandwich-style” dot blot assay employing both anti-Ac and mAb-29, in which the bacterial cells were concentrated by the anti-Ac pre-coated on the membranes and then probed with GfED-tagged mAb-29, as illustrated in Fig 5a. Serial dilution of anti-Ac IgG was assayed to determine the sensitivity of the test. The results showed that bacterial samples from inoculated leaves and *A*. *citrulli* Aac31 bacteria (10^8^ CFU/ml) can be captured by anti-Ac pre-coated on the NC membrane and detected by GfED-tagged mAb-29 with visible fluorescence. Moreover, NC membrane pre-coated with ~781 ng anti-Ac IgG could capture enough Ac bacteria which allowed subsequent detection with visible fluorescence. In contrast, *A*. *cattleyae* OAC1 (10^8^ CFU/ml) showed only weak signal in the detection, and was barely observable while the concentration of pre-coated anti-Ac IgG was lower than ~6.25 μg (Fig 5b). The result revealed the potential of this detection method that utilize both pre-coated polyclonal antibodies and GfED-tagged monoclonal antibodies to increase the ease of use of the assay. It should be noted that when high amounts of IgG were pre-coated on the membrane, the chance of false positive effects would increase which might be caused by excessive anti-Ac IgG molecules that could not be blocked by SpaED in the blocking step. Nevertheless, the results showed that the GfED-tagged antibody could be applied in practical use on the detection of Ac in infected field samples. Thus, plant-made GfED mediated by the BaMV-based vector could be employed as a helpful tool for the development of a convenient pathogen detection assay.

### Interactions among GfED proteins, antibodies, and antigens may lead to aggregations that could intensify the fluorescence

**Fig 6:**
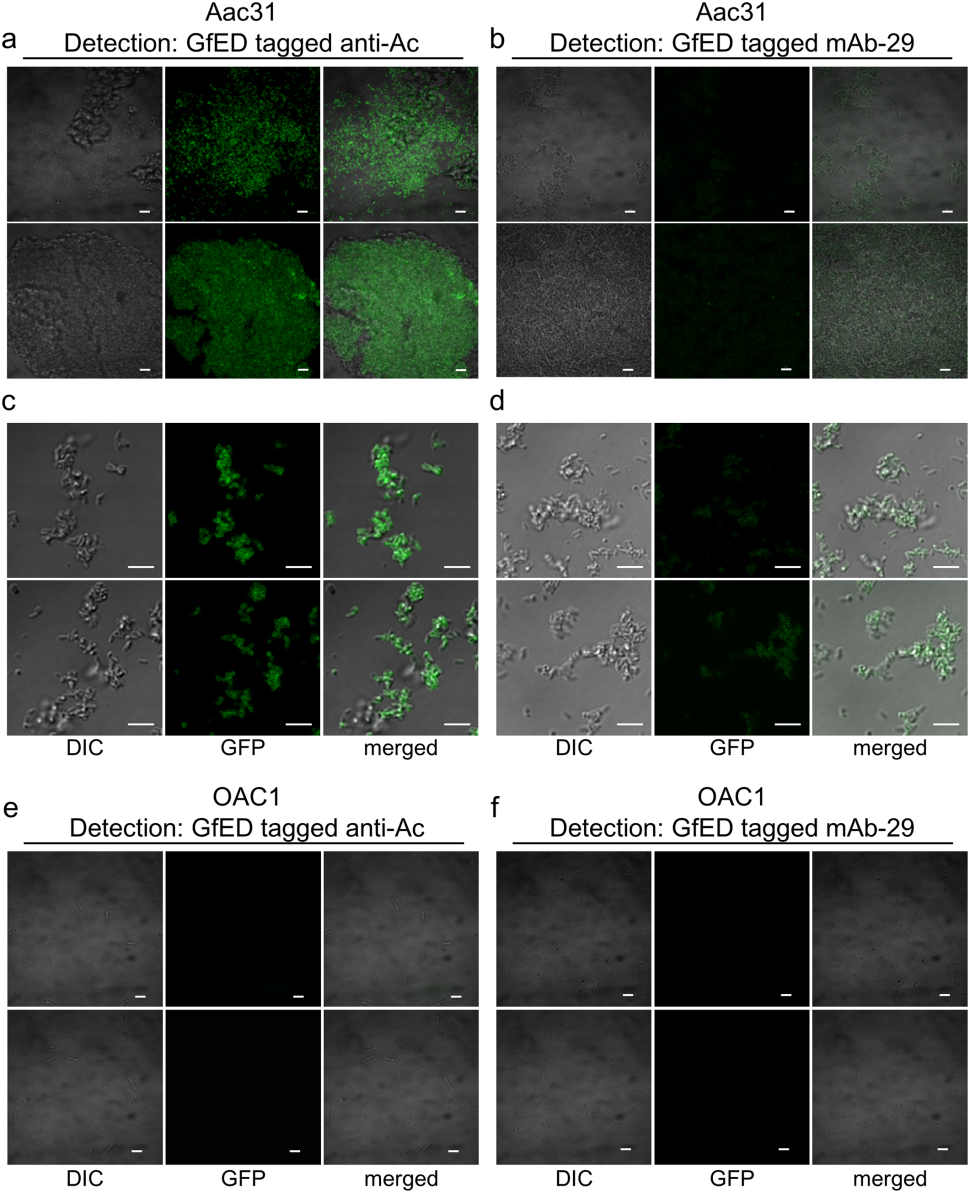
Confocal microscopy examination of the aggregation effect of GfED-tagged anti-Ac on the bacterial pathogen Aac31. The interactions between the Aac31 bacterial cells and anti-Ac antibody labeled with chimeric BaMV virions displaying GfED were examined by confocal fluorescence microscopy. *A*. *citrulli* Aac31 **(a-d)** or *A*. *cattleyae* OAC1 **(e, f)** (10^7^ CFU/ml) were mixed with GfED-tagged anti-Ac **(a, c, e)** or mAb-29 **(b, d, f)**, and shaken at 4°C for 1 hr, followed by centrifugation to collect the bacterial cells bound by the tagged antibody. After resuspension, the antibody-bacteria pellet were applied on a glass slide and examined by confocal laser scanning microscopy (Olympus FV1000, Tokyo). Fluorescent signals specifically co-localized with Aac31 cells. DIC, same areas examined by differential interference contrast; GFP, green fluorescence excited at 488 nm and detected at 525 nm; merged, green fluorescence and DIC merged micrographs. Scale bar, 2 μm.

It has been reported that coexpression of viral coat protein-fused fluorescent protein and wild type CP would result in CP dimers and CVPs with fluorescent protein on the surface [37]. We have also shown that expression of foreign proteins using the BaMV-based vector may result in the production of CVPs displaying the foreign proteins [16,17]. Since the “cleavage” efficiency of the FMDV 2a co-translational dissociation peptide (Fig 2) is not 100%, the resulting GfED fusion protein may contain the coat protein of BaMV, which may contribute to the affinity among GfED fusion proteins or the assembly of fluorescent CVPs. The aggregation of the GfED may further enhance the fluorescence intensity. Therefore, we examined the recognition of *A*. *citrulli* Aac31 by GfED-tagged anti-Ac (Fig 6a and 6c) or mAb-29 (Fig 6b and 6d) with confocal fluorescence microscopy. The results showed that the fluorescent signals were co-localized with *A*. *citrulli* Aac31 bacteria aggregation (Fig 6a–6d). In contrast, little or no fluorescent signals or the aggregation of bacterial cells were observed when *A*. *cattleyae* OAC1 bacterial cells were treated with GfED-tagged anti-Ac or mAb-29 (Fig 6e and 6f), although *A*. *cattleyae* OAC1 could be recognized relatively weakly by anti-Ac in dot blot assay (Fig 4d). The results showed that the GfED fusion proteins were specifically co-localized with *A*. *citrulli* Aac31 bacteria and the bacterial cells were aggregated by the GfED-tagged anti-Ac. This observation suggested that the GfED proteins may self-interact, possibly through the BaMV CP, to form aggregates with enhanced fluorescence intensity for the detection of pathogens by using fluorescence microscope. The assembly and characteristics of fluorescent CVPs were verified by the following studies.

### GfED proteins may self-assemble into virus-like particles (VLPs)

**Fig 7:**
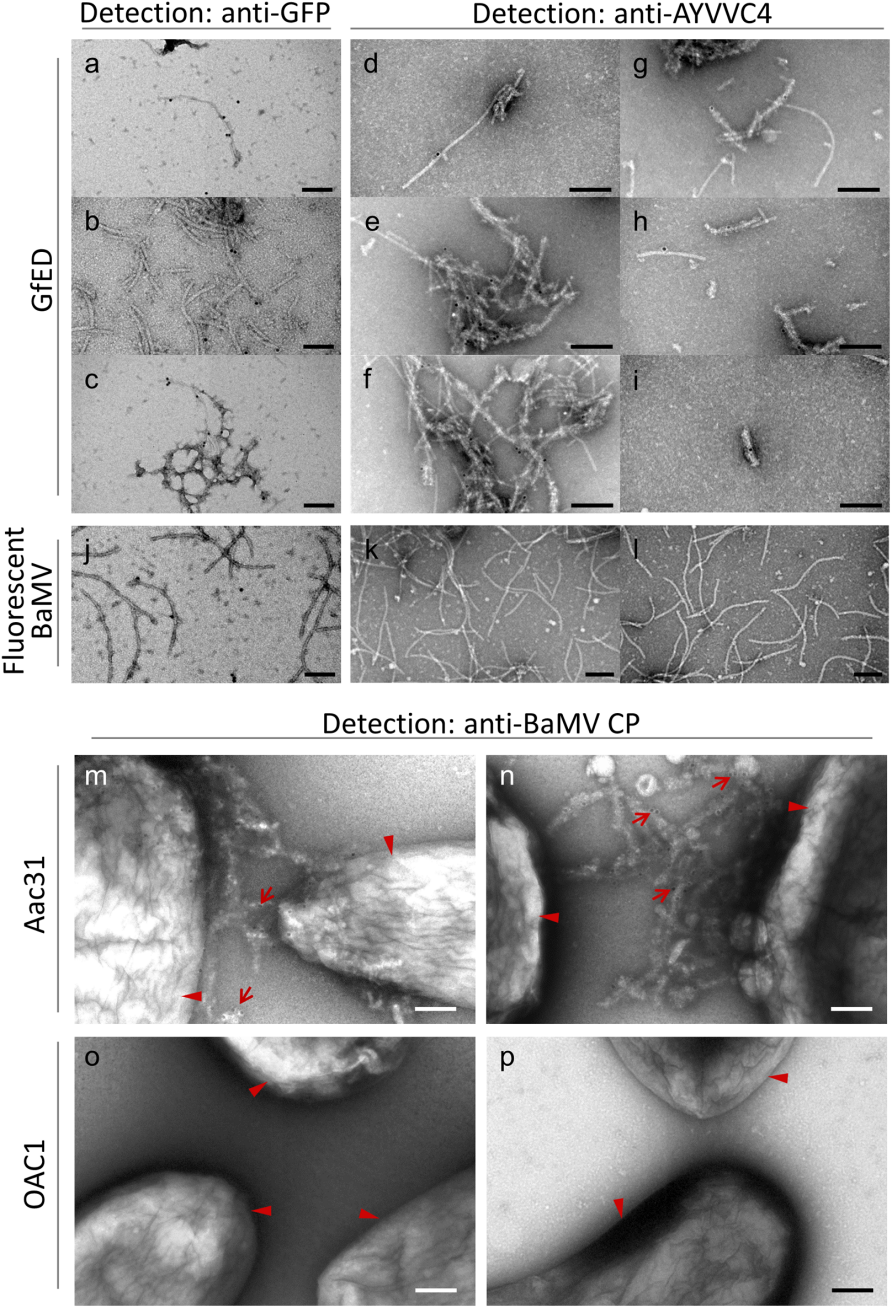
Examination of GfED aggregates and the interactions between Aac31 bacteria and GfED by transmission electron microscopy (TEM). **(a-l)** TEM examinations of GfED aggregates and fluorescent BaMV virions. Purified GfED aggregates **(a-i)** and fluorescent BaMV **(j-l)** were captured by anti-BaMV CP coated grids and detected using anti-Rb Gold conjugate linked anti-GFP **(a-c, j)** or anti-AYVVC4 **(d-i, k-l)** antibodies, as indicated on the top. **(m-p)** TEM examinations of the Aac31 **(m, n)** or OAC1 **(o, p)** bacterial cells treated with GfED-labeled anti-Ac antibodies and anti-Rb Gold conjugates. Aggregates of GfED virus like particles (VLPs) detected by anti-Rb Gold linked anti-BaMV CP were localized on Aac31 **(m, n)** but not on OAC1 **(o, p)** bacteria. Arrow head, bacterial cell; red arrow, gold nanoparticle on GfED VLP; scale bar, 200 nm.

The above results suggested the GfED fusion proteins could form aggregations with enhanced fluorescence. To analyze the nature of these aggregations, the fluorescent GfED aggregates were purified and applied on anti-BaMV CP linked grids for examination by transmission electron microscopy (TEM). Fluorescent BaMV virions from leaves inoculated with pCB-GFP2a-CP were used as a negative control without SpaED. The GfED aggregates and BaMV virions were immunolabeled using anti-GFP or anti-AYVVC4 linked anti-rabbit antibody gold-conjugates (anti-Rb Gold, with gold particles of 15 nm in diameter) to detect the GFP or SpaED domain, respectively. After the negative staining using uranyl acetate (UA), the samples were examined by TEM. It was confirmed that the sGFP and spaED domain can be found on the surface of GfED-displaying VLPs (Fig 7a–7c and 7d–7i, respectively). The sizes of GfED VLPs varied in length from approximately 200 to 600 nm and also varied in diameter from 15 to 30 nm. Furthermore, GfED VLPs tended to form entangled structure (Fig 7c, 7e and 7f) which may further result in the aggregation of Aac31 bacteria (Fig 6a and 6b). The surface of fluorescent BaMV virions was also shown to be decorated with sGFP but not SpaED (Fig 7j–7l). The BaMV viral particles were more uniform in size and more dispersed in the fields of observation. We further confirmed the interaction of antibody linked GfED and bacteria. Aac31 and OAC1 bacteria were applied to the grids and treated with anti-Ac linked GfED. The GfED above were further immunolabled using anti-BaMV CP linked anti-Rb Gold conjugates. The entangled GfED VLPs were shown to colocalize with Aac31 (Fig 7m and 7n, red arrows) but not OAC1 (Fig 7o and 7p). The gold particles located on OAC1 might be the result of the recognition of anti-Rb Gold to anti-Ac which was bound weakly to OAC1 bacteria. The results revealed the structure of GfED chimeric VLPs and verified the interaction between GfED and bacteria.

## Discussion

In this study, we constructed the GfED fusion protein as the antibody-labeling agent for the fluorescent detection technique. The BaMV-based vector [18] was employed for the practical production of GfED in *C*. *quinoa* plants. The GfED in the crude extracts from inoculated *C*. *quinoa* leaves could directly be applied to label monoclonal or polyclonal antibody molecules against specific antigens without further chemical treatment. We further devised a “sandwich-type” dot blot detection method, in which target bacteria were firstly concentrated by antibody coated NC membrane and then probed with GfED-tagged specific monoclonal antibody. Together, these results indicated that, in addition to the many detection methods for pathogens in plants have been reported [2], the GfED and the “sandwich-type” dot-blotting developed in the current study provides a useful alternative for the development of rapid detection tools.

The usages of GFP fused to full-length or partial Protein A (or G) as the fluorescent labeling agents have been described in several previous studies [5–8]. However, for practical applications, the cost for the production of such fluorescent labeling agents is one of the major concerns. In this regard, the usage of plants as “bio-reactors” for target protein production [9,10] in combination of the expression vectors based on plant viruses [11–13] offers an applicable alternative for the production of fluorescent antibody labeling proteins. In this study, we have adopted the BaMV-based vector previously developed in our group [18] for the practical production of GfED in *C*. *quinoa*. Furthermore, the GfED in the crude leaf extracts can be directly utilized as an antibody labeling agent without further purification process (as shown in Fig 3c and 3g). Thus, the GfED may serve as a useful option for the development of serological detection tools.

The incompletely blocking of SpaED binding sites on antibodies pre-coated on membranes and the interaction between SpaED and Fab region of IgG [38,39] might result in noise-signals as seen in Fig 5a. The reversible binding between SpaED and IgG [40,41] and the competition between GfED and SpaED peptide used as the blocking reagent could decrease the signals of target detection. The use of ZZ domain, an artificial domain B mutant of SpA with reduced affinity to Fab region of IgG [42,43], or the chemical conjugation between IgG-binding domain of GfED and the corresponding IgG may provide a solution to reduce the interference. Nevertheless, in the current format, GfED offered sufficient sensitivity and specificity for the detection of two plant pathogens, Ac and BaMV.

The “cleavage” efficiency of the FMDV 2a co-translational dissociation peptide [44] is known to be affected by the fusion peptide sequence [45,46]. In this study, *spaED* gene was ligated to 5’-end of sGFP coding sequence for the construction of pCB-spaED-GFP2a-CP (harbored in clone N6). In contrast, in the construct pCB-GFP-spaED2a-CP (harbored in clone C31), the *spaED* sequence was ligated to the 3’-end of sGFP coding sequence with no spacer in between. Inoculation of N6 on *C*. *quinoa* resulted in fewer lesions on inoculated leaf (Fig 2c) and also less production of fusion protein (Fig 3b–3d), compared to those inoculated by C31. It might be a result of the differences in the “cleavage” efficiency of the constructs that led to the interference on the proper folding or assembly of CP [47,48]. The inclusion of the FMDV 2a peptide in the fusion construct resulted in the production of two types of GfED (Fig 3i), with or without the fusion of BaMV CP. CP and sGFP fusion proteins derived from a construct containing FMDV 2a peptide have been shown to be able to assemble into fluorescent CVPs [37]. In our previous studies, the presence of both the recombinant proteins and free-form BaMV CP facilitated the assembly of CVPs [15–17]. In this study, the presence of free-form BaMV CP and “un-cleaved” form of GfED may have facilitated the assembly of CVPs, enhancing the intensity of fluorescence and providing more binding sites for IgG molecules, which may increase the sensitivity of GfED when used as an antibody-labeling agent for serological detection. However, this possibility should be verified with further studies.

Both fusion proteins produced by clones N6 and C31 were shown to exhibit the affinity to IgG molecules, and the fusion proteins from both clones in crude extracts of inoculate *C*. *quinoa* leaves were mixed and shown to be a competent fluorescent antibody-labeling agent for detection assays. The crude lysate of *E*. *coli* BL21 (DE3) cells which contained SpaED protein was used as a blocking agent for detection assays in which antibodies were pre-coated on the NC membrane. Thus the cost for the production of reagents used in the “sandwich-type” dot blot assay was considerably lowered, which complies with the requirement for practical applications.

The *A*. *citrulli* Aac31 cells were observed as aggregates bound by GfED-tagged anti-Ac, suggesting the interactions among GfED fusion proteins (Fig 6). It is possible that the aggregation effect was contributed by the affinity among the un-cleaved form of BaMV coat protein subunits in GfED fusion protein or as a result of the association of fluorescent CVPs with multiple IgG-bound GfED fusion proteins. The clustering of pathogens and GfED fusion proteins could improve the visibility under microscope and fluorescent CVPs might provide more intense fluorescent signals for identifying target pathogens. The constructs used in this study allowed the production of VLPs assembled from both wild-type CPs of BaMV and sGFP-SpaED fused BaMV CPs. Each VLP may display multiple copies of sGFP-SpaED, potentially providing the enhancement of green fluorescence intensity, even when only one VLP is bound to the target antibody. In addition, the multiple copies of SpaED allowed one VLP to interact with multiple antibodies, which in turn may bind to multiple target antigens, leading to the formation of aggregates of GfED-antibody-antigen complexes and further enhancement of the fluorescence intensity, as shown in Figs 6 and 7. Thus, our approach provided two levels of signal enhancement: (i) the enhancement through the assembly of multiple GfED into each VLP; and (ii) further enhancement through the formation of GfED-antibody-antigen aggregates. In addition, the use of plants as the production platform provides the ease in scaling up and maintenance. The direct application of crude saps from leaves expressing GfED further eliminated the need for tedious protein purification processes.

A competent fluorescent detection depends not only on the reagents, but also devices for observation of the results. In this study, the fluorescence of the results was visualized by inexpensive homemade, handheld devices (S1 Text and S1 Fig), consisting of a modified LED flashlight with blue light of 450 nm wavelength as an excitation source and a transparent orange acrylic plate as the filter. The results were directly taken using regular digital camera through these devices, demonstrating their capability and portability for on-site detection purposes. LED illumination with different wave lengths (420–485 nm) and the transparent acrylic plates with different colors have been tested with varying degrees of success. Future improvements on the combinations of excitation light source and filter color might provide further enhancement on the sensitivity of this fluorescent detection technique.

In conclusion, we have adopted a BaMV-based vector for the practical production of a fluorescent antibody-labeling agent, GfED, in *C*. *quinoa* plants, and demonstrated the application in immunofluorescence detection techniques. The materials and devices involved were relatively low-cost and easy for mass manufacturing. The GfED and “sandwich-type” dot blot assay may offer useful alternatives for the development of serological detection tools for pathogens or other targets.

## Supporting information

S1 Text Homemade, handheld blue light illumination devices. (DOCX)

S1 Fig Handheld blue light illumination devices for observation of sGFP signals on leaves and NC membranes. (TIF)

S2 Fig Detection of anti-Ac IgG by dot blot analysis using GfED recombinant proteins in crude sap. (TIF)

S3 Fig Original, uncropped gel image of Fig 1b. (TIF)

S4 Fig Original, uncropped gel images of Fig 2b. (TIF)
