# Supplementary material for: Production of fluorescent antibody-labeling proteins in plants using a viral vector and the application in the detection of Acidovorax citrulli and Bamboo mosaic virus
Source: PLoS One. 2018 Feb 6;13(2):e0192455. doi: 10.1371/journal.pone.0192455 (PMC5800667; doi:10.1371/journal.pone.0192455)
Supplement: S1 Text — (DOCX) [file pone.0192455.s001.docx]

**Supporting Information Text and Legend to Supplementary Figures**

**S1 Text. Homemade, handheld blue light illumination devices**

The fluorescent protein sGFP could be excited by light at 365 nm (UV light) and 485 nm (blue light), resulting in the emission of green fluorescence at 508 nm. To facilitate the on-site monitoring and detection of green fluorescence on GfED-expressing leaves (Fig. 2c) or test membranes (Fig. 4b, d, and 5b), two handheld illumination devices were made in the laboratory. For the concerns of portability, light intensity, and stability of excitation wavelength, a high powered device, designated Blue Gear 132 (BG132), was created (Fig. S1a). To reduce production cost, BG132 were made of recycled materials, including bike gears, LED bulbs (450 nm), switches, cooling fans, and Li-ion batteries. The bike gears provide robust backbone of the device and better cooling of LED bulbs. The installation of 132 blue-light LED bulbs provides sufficient excitation light (2133 lm, measured at a distance of 50 cm) both in the fields and in the laboratory for the present study. In addition, 2 white-light LED bulbs allowed the observation of leaf morphology in the dark. BG132 was equipped with 4 18650 Li-ion batteries, providing non-stop operations for up to 50 min. BG132 is able to illuminate the fluorescent lesions on plants in batch mode from a distance (200 cm) (Fig. S1b and c, respectively), and also the sGFP signals within acrylamide gel (Fig. 3e) or on NC membrane (Fig. 4b, d, and 5b). For the detection of fluorescent signals on small pieces of NC strips on site, a mini device, designated Blue Chip 6 (BC6), with better portability was developed (Fig. S1d). With a compact size (10 cm × 4.5 cm × 1 cm; 26.1 g), BC6 consisted of 6 blue-light LED bulbs which were directly driven by a CR2032 Li cell. The fluorescent signals on NC strip could be detected using BC6 in relatively dark place under a transparent orange acrylic lid or plate (Fig. S1e) as filters. The performance of BC6 was shown in the detection of BaMV virions (Fig. S1e) which were examined using BG132 as well (Fig. 4b).

**Figure legends**

**S1 Figure. Handheld blue light illumination devices for observation of sGFP signals on leaves and NC membranes. (a)** Blue light illumination device BG132 was made of recycled materials as indicated. BG132 was equipped with 132 blue light LEDs, sufficient to excite the emission of green fluorescence of sGFP on leaves in batches **(b)** or at a distance **(c)**. **(d)** The credit card sized Blue chip 6 (BC6), designed for easy portability, was suitable for examination of the sGFP signal on NC strips. **(e)** Performance of BC6. Wild type BaMV virions with different concentrations as indicated were dotted on a NC strip, reacted with GfED-linked anti-BaMV CP as shown in Fig. 4b, and examined using BC6.

**S2 Figure. Detection of anti-Ac IgG by dot blot analysis using GfED recombinant proteins in crude sap.** Various amounts of anti-Ac IgG, as indicated, were loaded on nitrocellulose membranes, which were then blocked with 5% milk. The binding sites on IgG were further either blocked by SpaED protein (SpaED-blocked) or not blocked (non-blocked), as indicated. The membranes were then overlaid with crude sap containing GfED (1 g fresh leaf in 2.5 ml) for 1 hr. The results were observed under 450 nm blue light excitation with orange filter.

**S3 Figure. Original, uncropped gel image of Figure 1b.** The cropped region used for Figure 1b (left panel) is indicated by the red box. The condition for electrophoretic analysis is as described in the legend to Figure 1b.

**S4 Figure. Original, uncropped gel images of Figure 2b.** The cropped regions used for Figure 2b (upper panel) are indicated by the red boxes. The condition for electrophoretic analysis is as described in the legend to Figure 2b.
