# Supplementary figures and images for: Production of fluorescent antibody-labeling proteins in plants using a viral vector and the application in the detection of Acidovorax citrulli and Bamboo mosaic virus
Source: PLoS One. 2018 Feb 6;13(2):e0192455. doi: 10.1371/journal.pone.0192455 (PMC5800667; doi:10.1371/journal.pone.0192455)

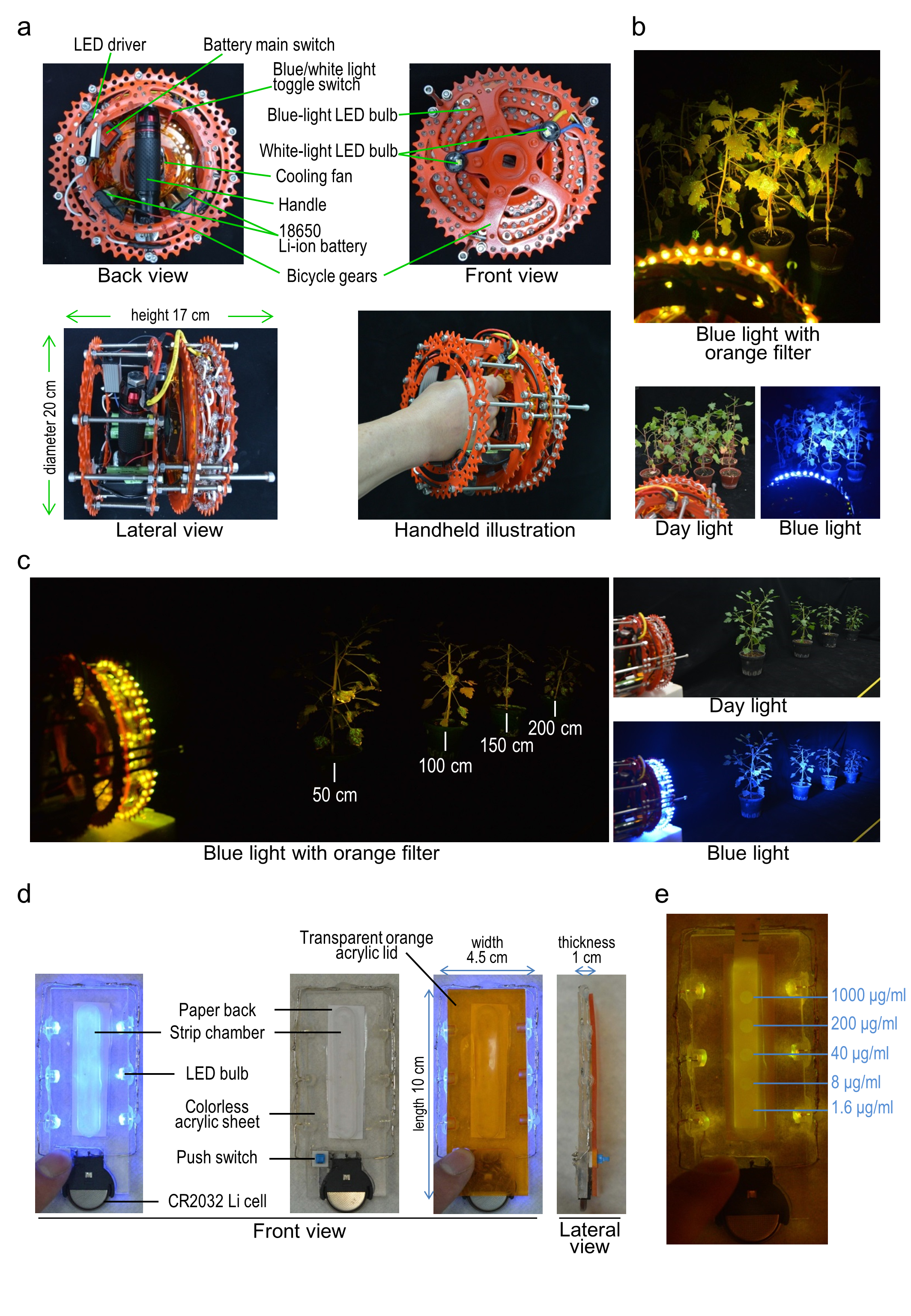

Supplement: S1 Fig — (TIF) [file pone.0192455.s002.tif]

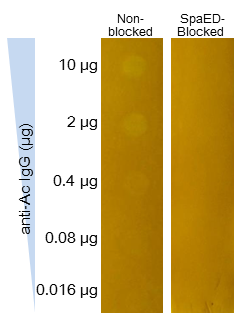

Supplement: S2 Fig — (TIF) [file pone.0192455.s003.tif]

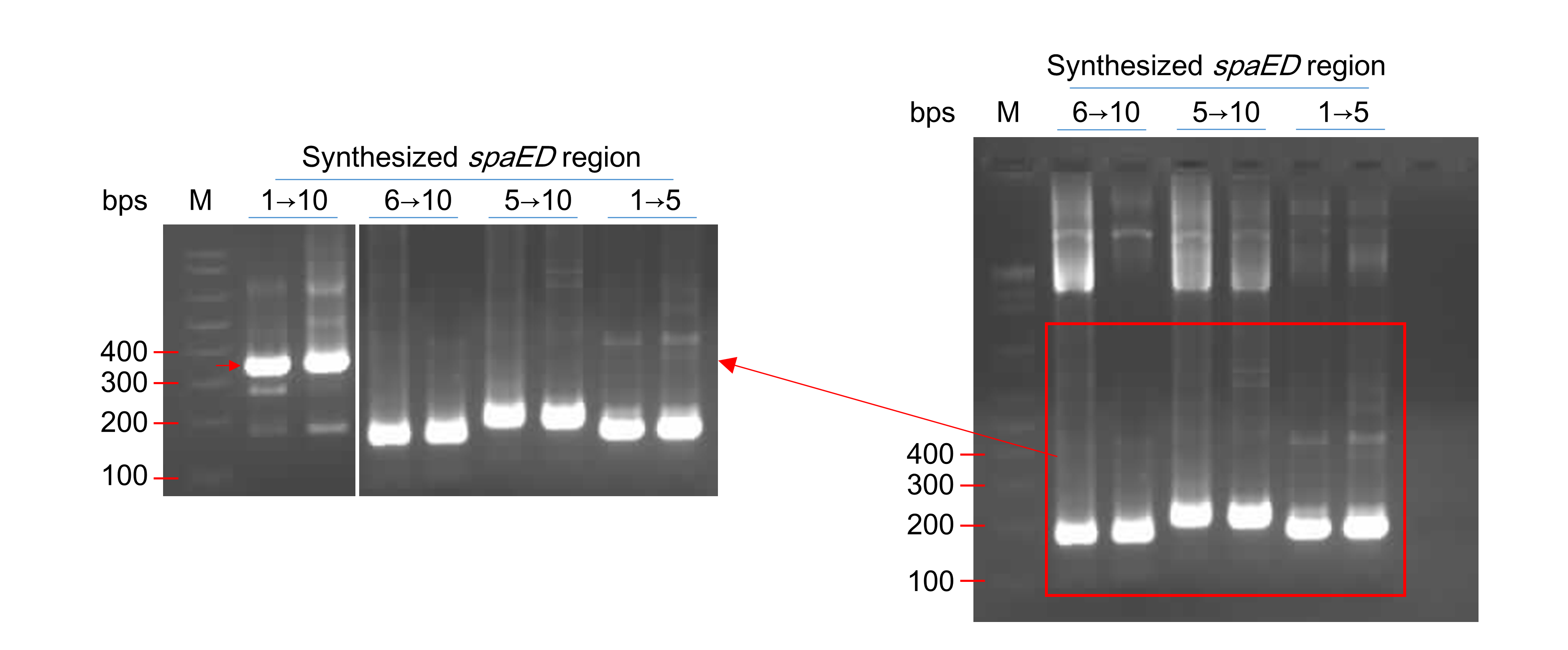

Supplement: S3 Fig — (TIF) [file pone.0192455.s004.tif]

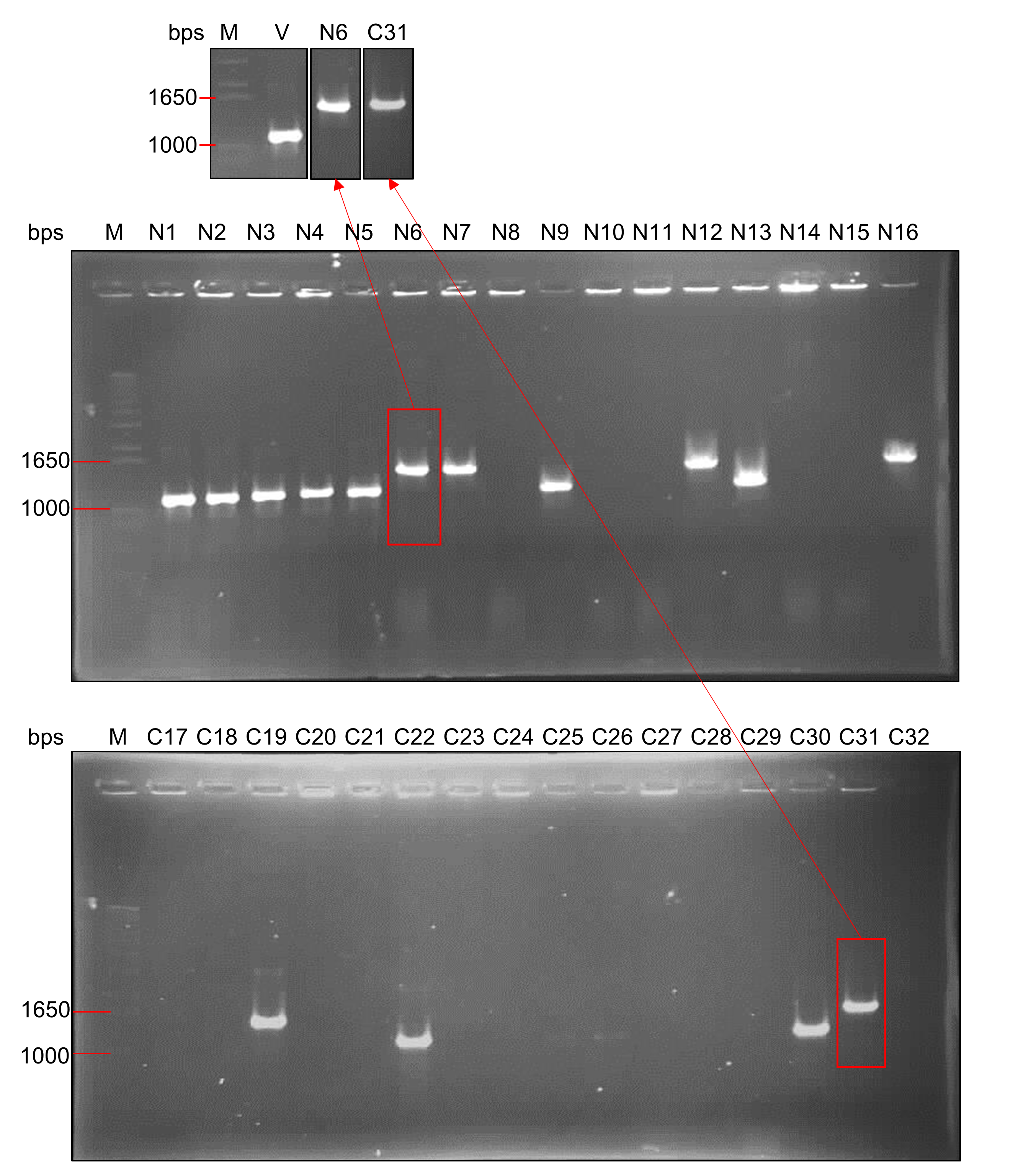

Supplement: S4 Fig — (TIF) [file pone.0192455.s005.tif]
